# A glioneuronal tumor with *CLIP2-MET* fusion

**DOI:** 10.1038/s41525-020-0131-6

**Published:** 2020-06-03

**Authors:** Tamrin Chowdhury, Yeajina Lee, Sojin Kim, Hyeon Jong Yu, So Young Ji, Jeong Mo Bae, Jae Kyung Won, Joo Heon Shin, Daniel R. Weinberger, Seung Hong Choi, Chul-Kee Park, Jong-Il Kim, Sung-Hye Park

**Affiliations:** 1 Department of Neurosurgery, Seoul National University College of Medicine, Seoul National University Hospital, Seoul, 03080 Korea; 2 Department of Biochemistry and Molecular Biology, Seoul National University College of Medicine, Seoul, 03080 Korea; 3 Genomic Medicine Institute, Medical Research Centre, Seoul National University, Seoul, 03080 Korea; 4 Department of Pathology, Seoul National University College of Medicine, Seoul National University Hospital, Seoul, 03080 Korea; 5 Lieber Institute for Brain Development, Johns Hopkins Medical Campus, Baltimore, MD 21205 USA; 6 Department of Radiology, Seoul National University College of Medicine, Seoul National University Hospital, Seoul, 03080 Korea

**Keywords:** Cancer genomics, Oncogenesis

## Abstract

We report a case of glioneuronal tumor (GNT) with a discovery of novel gene fusion of *CLIP2-MET* resulting from aberrant chromosome 7 abnormalities. We executed an elaborate genomic study on this case including whole-exome sequencing and RNA sequencing. Genomic analysis of the tumor revealed aberrations in chromosomes 1 and 7 and a *CLIP2-MET* fusion. Further analysis of the upregulated genes revealed substantial connections with MAPK pathway activation. We concluded that the chromosome 7 abnormalities prompted *CLIP2-MET* gene fusion which successively leads to MAPK pathway activation. We deliberated that MAPK pathway activation is one of the driver pathways responsible for the oncogenesis of GNT.

## Introduction

Glioneuronal tumor (GNT) is one type of biphasic central nervous system (CNS) tumor which exhibits both glial and neuronal immunohistological characteristics^1^. In the WHO classification of CNS tumors, they are given a specific category under the heading of neuronal and mixed neuronal-glial tumors which consists of diverse morphological neuroepithelial tumors with neuronal and/or glial differentiation^2,3^. Most of the tumors under the neuronal and mixed neuronal-glial tumors are benign and usually shows good prognosis although some exceptions exist^4^. Controversies still exist, however, concerning the spectrum of glial tumors with neuronal differentiation, and their sub-classification is still under active investigation based on genetic and epigenetic backgrounds identified^5,6^. Among those few studies on genetic events in the GNT category to date, *NTRK1* or *BRAF* gene fusions are found in the subset of GNTs^5^. Recent study identified the signature *PRKCA* gene fusions and *FGFR1* gene mutation in papillary glioneuronal tumors (PGNT)^6–9^. However, there are still blanks in genetic events that need to be investigated in GNTs.

There has been evidence identifying mesenchymal–epithelial transition factor (*MET*) related gene fusions as an oncogenic driver in glioma progression by upregulating mitogen-activated protein kinase (MAPK) signaling pathways^10–13^. Among them, CAP-GLY-domain-containing linker protein 2 (*CLIP2*) with MET fusion was identified in a pediatric glioblastoma cell line and infantile high-grade glioma^12,13^. However, no reports have yet mentioned *CLIP2-MET* fusion in adult lower-grade gliomas. Here we present a case of a 30-year-old woman diagnosed with GNT harboring *CLIP2-MET* fusion and copy number alteration of chromosome 7.

## Results

**Fig. 1:**
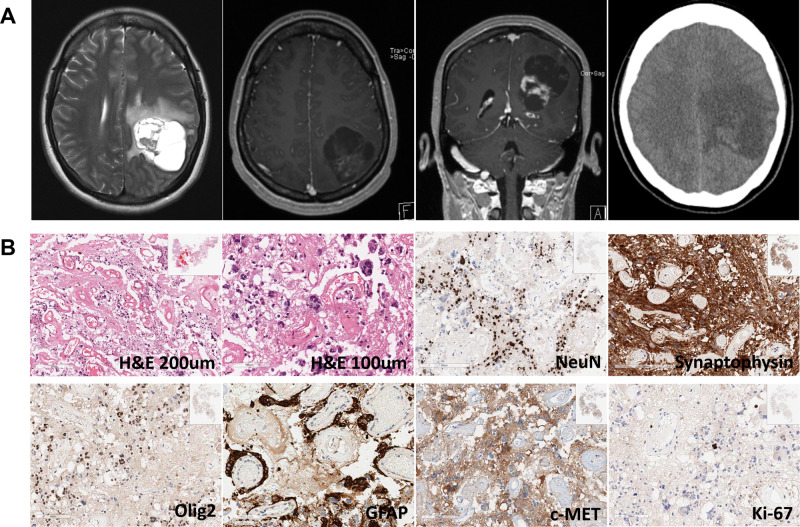
Radiological and histological features of a 30-year-old glioneuronal tumor (GNT) case. **a** Magnetic resonance images show multi-cystic mass in left parietal lobe with peritumoral edema. Scanty enhancement is observed in the solid portion of the mass. No calcification is identified in the computed tomography. **b** The tumor shows well-developed blood vessels with perivascular hyalinization with sheet of tumor cells between the blood vessels (H&E bar: 200 μm). Atypical hyperchromatic or multiple nuclei are observed, which are possibly degenerative atypia produced by long-standing slow growing nature of the tumor (H&E bar: 100 μm). Immunohistochemical studies reveal positive tumor cell nuclei for NeuN antibody (bar 200 μm), diffuse strong positivity in tumor cells for synaptophysin (bar 200 μm), focal positivity for Olig2 (bar: 100 μm), focal positivity for GFAP (100 μm), diffuse positivity for c-MET (bar: 100 μm), and low Ki67 labeling index of 0.4% (bar: 100 μm).

A 30-year-old woman was admitted for surgery with a complaint of intermittent dysphasia and right arm pain. General neurological examination before surgery showed no abnormality, except for the moderate attention deficit in the neuro-cognitive function test. Magnetic resonance images (MRI) showed a mixed solid and cystic mass in the left parietal lobe (Fig. 1a). Total surgical resection was achieved without any newly developed deficits. The histological diagnosis using immunohistochemistry (IHC) studies was compatible with GNT (Fig. 1b). No evidence of isocitrate dehydrogenase 1 (*IDH1*) mutation nor *BRAF* mutation was observed in IHC studies. Fluorescence in situ hybridization studies revealed no chromosomal 1p/19q co-deletion, and no amplification for *EGFR* as well as for c-MET. After surgery, no adjuvant treatment was applied, and the patient remained a complete remission state for 7 years.

We collected blood and tumor sample from the patient during the surgery with appropriate written informed consent. After DNA and RNA extraction from the samples, whole-exome sequencing (WES), RNA sequencing (RNA-seq), and methylation sequencing (Methyl-seq) was done utilizing current Illumina sequencing platforms. Somatic mutation, germline mutation, and copy number variations were detected from the WES data using the Mutect2, Haplotypecaller, and CNVkit programs correspondingly^14,15^.

**Fig. 2:**
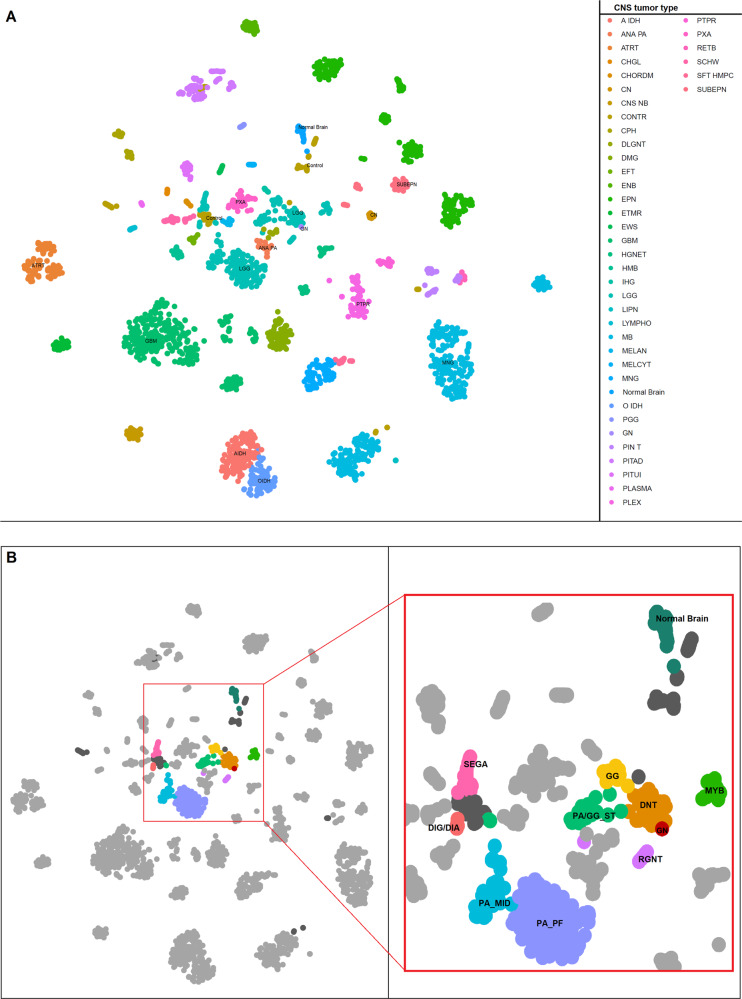
t-SNE map showing the categorization of the glioneuronal tumor (GNT) and normal brain samples with public CNS tumor data. **a** t-SNE map showing cluster of different CNS tumor groups with the GNT sample clustering with the low-grade glioma samples and the normal brain sample with the control group. **b** t-SNE map with the GNT and normal brain sample shown in magnified view with detailed classification of the low-grade gliomas. GNT sample is clustered with the DNT samples specifically.

We used the Methyl-seq data to confirm the diagnosis of the tumor as GNT by comparing the data with previously published epigenetic classifier of CNS tumors using t-distributed stochastic neighbor embedding (t-SNE) analysis^16^. The detailed information on the analytic process is described in the Methods section. In the t-SNE map our GNT sample was grouped with the low-grade glioma (Fig. 2a) and dysembryoplastic neuroepithelial tumor (DNT) group (Fig. 2b) which is one of the subclass of the neuronal and mixed neuronal-glial tumors in the 2016 WHO CNS tumor classification^3^. To confirm the validity of the bioinformatical process of using Methyl-seq data for methylation classifier, we employed normal brain samples encompassing same analytical process, and we could confirm that they are mapped with control group in t-SNE analysis (Fig. 2a, b).

**Fig. 3:**
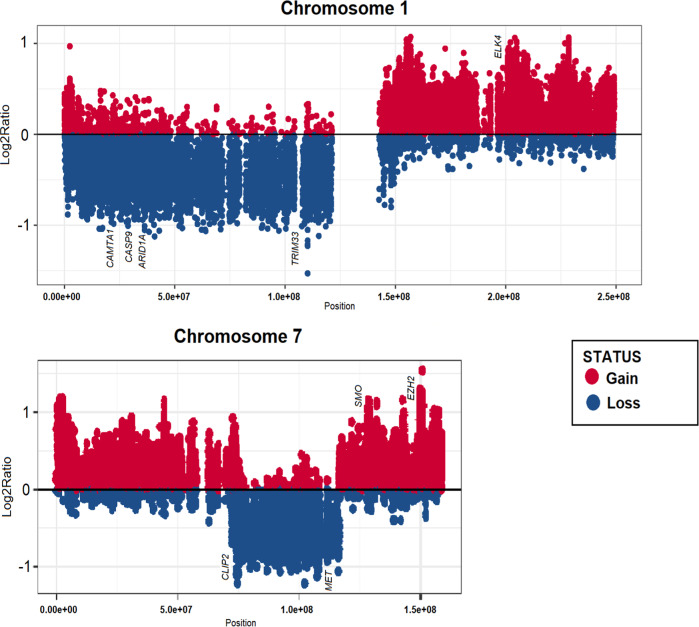
Copy number abnormalities and somatic mutations in the reported case of glioneuronal tumor (GNT). Both chromosomes 1 and 7 shows a significant amount of copy number gains and losses compared to the other chromosomes.

The genome-wide copy number analysis revealed partial losses and gains in the chromosome 1 and 7, encompassing tumor suppressor genes such as *TRIM33, CAMTA1, CASP9, ARID1A*, and oncogenes such as *ELK4, SMO, EZH2* which were annotated in COSMIC, the catalog of somatic mutations in cancer (Fig. 3, Supplementary Data Set 1). Somatic mutations from WES analysis were filtered with 1000 g, ExAC, gnomAD (allele frequency < 0.01) databases focusing on the exonic and splicing regions. It included genes with 25 nonsynonymous SNVs (*PRAMEF2, KANK4, ARHGEF11, LMOD1, KCNK12, KCNK12, EML6, LRTM1, FAM81B, F12, DSP, DOPEY1, CNBD1, MUC5B, TRIM49, EXOSC8, SPOP, KLHL14, AP3D1, ZNF208, FXYD5, PPP2R3B, USP11, HS6ST2, TREX2*), 2 synonymous SNVs (*SLC25A34, MYOC*), 1 stop-gain (*ZNF536*), 1 frameshift deletion (*ZACN*), and 1 frameshift insertion (*WASHC4*) (Supplementary Data Set 1). No germline driver mutations were found. The tumor mutation burden ratio is 0.48.

**Fig. 4:**
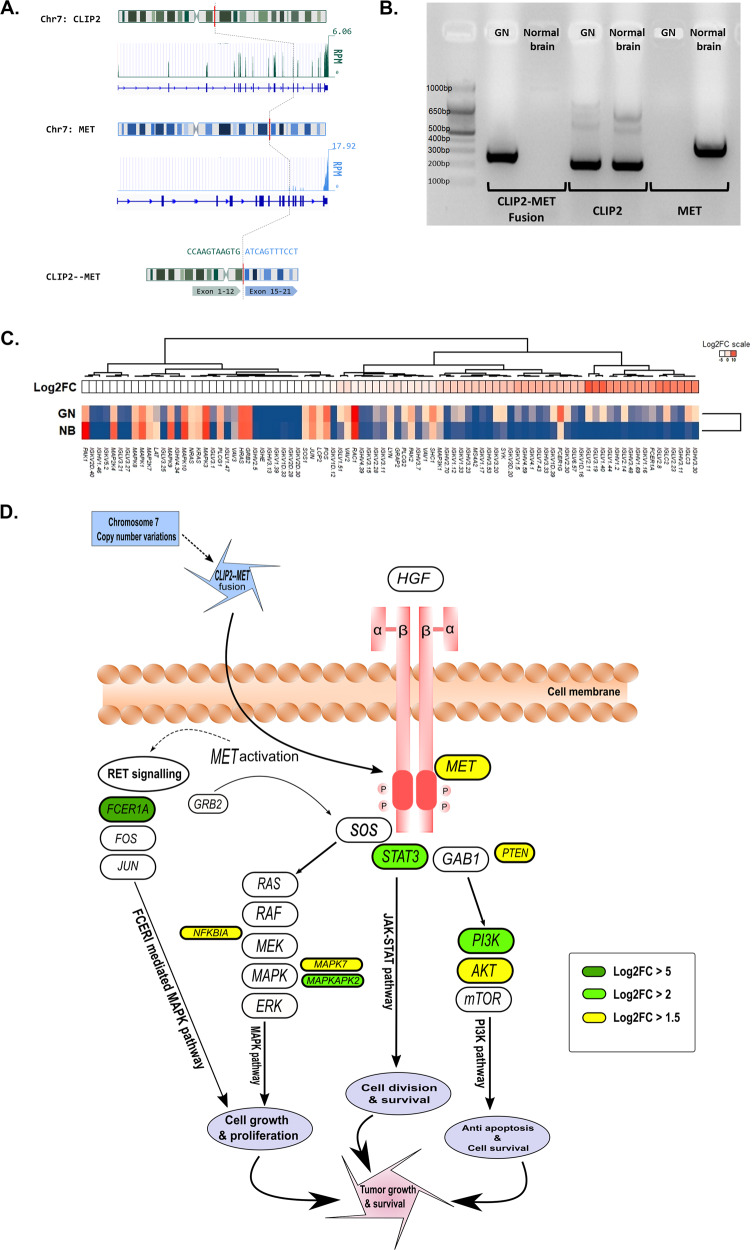
*CLIP2-MET* fusion activates the MAPK pathway cascades in the glioneuronal tumor (GNT). **a** Schematic diagram of the *CLIP2-MET* fusion showing the fusion point with corresponding RNA expression values. **b** RT-PCR with the fusion gene primer showing clear bands in the GNT sample and no bands in the normal brain sample. **c** Genes associated with the FCERI-mediated MAPK activation pathway upregulated in the GNT compared to normal brain. **d** Schematic diagram showing *CLIP2-MET* fusion gene activating the MAPK pathway cascade leading to tumor growth and survival.

From the RNA-seq data, we conducted the gene expression and fusion gene analysis using the RSEM and Star Fusion package subsequently^17,18^. We excluded the fusions that had zero spanning reads and/or sharing the same spanning read counts and junction read counts with one gene. We only considered the fusion as valid if both the genes were a protein coding gene. With these criteria, we detected an inframe intrachromosomal *CLIP2-MET* fusion in chromosome 7. The spanning read count of this fusion was 50 out of 99 junction read counts. The exon 1–12 of *CLIP2* was fused with exon 15–21 of the *MET* kinase domain (Fig. 4a). The transcript reads were not detected before *CLIP2* fused to the *MET*. The reads were expressed after the breakpoint. The expression of the *CLIP2-MET* fusion gene in GNT was confirmed by RT-PCR (Fig. 4b). The expression of the *MET* gene, which can be expressed in the normal brain, was affected in the tumor due to the fusion as no bands were detected in the GNT sample, while clear band was detected for the fusion gene. We also verified the gene expression counts and the transcript reads were not detected before *CLIP2* fused to the *MET*. The reads were expressed after the fusion breakpoint. This explains the higher expression of the *MET* gene in the GNT sample overall. Due to the lower transcript counts at the fusion site, the *CLIP2-MET* fusion specific primer of the *MET* gene did not produce any bands. But after the fusion point, the transcript reads were expressed and resulted in a high expression of *MET* in the GNT sample. Next, we investigated the RNA expression values of the GNT sample with those of 36 normal brain frontal cortex^19,20^. We compared the average expression of the normal brain samples and the GNT sample and determined the Log2 fold change (Log2FC) ratio. We extracted the highly upregulated (Log2FC ≥ 5) and downregulated (Log2FC ≤ −5) genes (Supplementary Data Set 1) and performed a gene set pathway enrichment analysis with gProfiler^21^. From the upregulated gene lists the pathways that were significantly enriched included *FCERI-*mediated MAPK activation and many immune systems-related pathways such as antigen binding, complement activation, humoral immune response, and B cell-mediated immunity (Supplementary Data Set 1). The downregulated genes enriched pathways included GPCR ligand binding and cAMP signaling (Supplementary Data Set 1). We then investigated the genes associated with the FCERI-mediated MAPK activation pathway and compared the expressions of these genes in our GNT and normal brain samples. We discovered that more than half of the genes associated with the *FCERI*-mediated MAPK activation pathway were upregulated in the GNT sample compared to the normal brain samples (Fig. 4c). Therefore, we contemplated that in this GNT case, chromosome 7 abnormalities aided the CLIP2-MET fusion which successively upregulated genes such as *MET, FCER1A, MAPKAPK2, MAPK7, NFKB1A,* etc. promoting MAPK pathway activation and inducing the tumorigenesis (Fig. 4d).

## Discussion

We performed genome-wide profiling for the case with typical histological characteristics of GNT. To further explore the diagnosis of the tumor, we compared the selected most variable methylation CpG sites of the case with the public methylation data of CNS tumors. The t-SNE analysis of our tumor and normal brain showed their class position at DNT and control of the public methylation classifier data, respectively^16^. The key genomic alterations in this case were found to be the copy number alterations in chromosomes 1 and 7 and the *CLIP2-MET* fusion gene. Structural alterations in chromosome 7p were reported previously in GNT like ganglioglioma, DNT, and PGNT^22,23^. The most interesting finding in the present case is that the discovery of the *CLIP2-MET* fusion gene. To our knowledge this is the first reporting of a *CLIP2-MET* gene fusion in a GNT tumor heretofore. The *CLIP2-MET* fusion gene has been identified to have oncogenic potential in pediatric glioblastomas^13^.

Crossroad of all the identified aberrant genome changes in the literature and the present study is the activation of mitogen-activated protein kinase (MAPK) pathway. It has been reported that fusions involving *MET* gene such as *CLIP2-MET* can lead to activation of the MAPK pathway^24,25^. Both *FGFR1* gene mutation and *SLC44A1-PRKCA* gene fusion had been reported to play a role in the activation of the MAPK pathway^7,8^. Therefore, it seems that MAPK pathway activation is the key process of the GNT oncogenesis. Many studies have reported that the MAPK pathway is one of the driving oncogenic pathways in low-grade gliomas. About 80% of diffuse leptomeningeal GNTs in children are reported to harbor gene mutations that lead to MAPK pathway activation^26^. However, 20–60% of all gangliogliomas with *BRAF* mutation revealed activation of the MAPK pathway^26^. It is also reported that activation of the MAPK pathway is observed in 90% of pilocytic astrocytomas as a single pathway disease^27^. Chromosome 7 partial deletion giving rise to BRAF gene fusions has been reported as the leading cause of MAPK pathway activation in pilocytic astrocytomas and other low-grade gliomas^28,29^.

In our study we found that many of the highly upregulated genes in our GNT sample were directly associated with MAPK pathway activation. We also found that many immune pathways were enriched with the upregulated genes in GNT. It was previously reported in multiple studies that MAPK pathway activation subsequently leads to numerous immune pathway activation and is one of the main factors of creating an immunosuppressive tumor microenvironment^30,31^. On the other hand, the downregulated genes in GNT were associated with pathways like GPCR ligand bindings and cAMP signaling pathways, both of which are reported to regulate the MAPK pathways negatively^32,33^. Some other significant genes like *PIK3CA, AKT1, PTEN, STAT3*, etc. were also upregulated in our study. These are key genes of pathways like PI3K signaling and JAK–STAT signaling and MAPK pathways are cross-linked with these pathways in various studies stating that activation of one pathway consequently leading to activation of the others^34,35^. All these results and information led us to the conclusion that MAPK pathway activation is the key oncogenic driver activated by chromosome 7 abnormalities and *CLIP2-MET* fusion in this GNT case. The findings in the present study will broaden the knowledge of GNT and will give insights into the development of treatment targeting the MAPK pathway.

## Methods

### Sample collection

This study was approved by the institutional ethics committees of Seoul National University Hospital. Patient sample was obtained after receiving written informed consent for research purpose including genetic studies in accordance with its institutional review board. After tumor removal, the tumor tissue was snap frozen with liquid nitrogen and then stored at −80 °C. Blood was also collected from the patient at the time of tumor removal and WBC was isolated from the blood and stored at −80 °C

### DNA and RNA extraction

DNA was extracted from the frozen tumor tissue and WBC samples with Qiagen QIAamp DNA mini kit (Qiagen, Valencia, CA) and RNA was extracted from the frozen tumor tissue with RNeasy Lipid Tissue Mini Kit (Qiagen, Valencia, CA) following the manufacturer's protocol. The extracted DNA and RNA were then sent to Macrogen, Korea for WES and RNA-seq.

### WES and RNA-seq

After quality control of the samples sequencing library was prepared by random fragmentation of the DNA or cDNA followed by 5′ and 3′ adapter ligation. Library preparation was done using the SureSelectXT library prep kit for WES and the TruSeq standard mRNA LT sample prep kit for RNA-seq. WES and RNA-seq were performed using the Illumina platform. The generated BCL binary was then converted into raw FASTQ files utilizing Illumina bcl2fastq package.

### Methyl-seq and CpG analysis

After quality control of the DNA samples, library preparation was done using the SureSelectXT Methyl-Seq Target Enrichment System for Illumina Multiplexed Sequencing Version D0, July 2015 protocol with SureSelect Methyl-Seq library prep kit. Methyl-seq was executed utilizing Illumina platform and the raw FASTQ files were generated by Illumina bcl2fast package. Bowtie2 (ver. 2.2.7) was used to align the read to the GRCh37 (ref. ^36^). Bismark (ver. 0.20.0) was used to calculate methylated and unmethylated reads^37^. The methyl ratio was calculated manually. The methylated reads were divided by the total reads, which is the sum of the unmethyl and methyl ratio of both strand.

The current GNT case, 36 samples of normal brain data, and 2801 samples of Capper et al. data were used for t-SNE analysis applying the Rtsne package^38^. The platform of the methylation data of our GNT case and the Capper et al. data were different. GNT case platform was targeted bisulfite sequencing and Capper et al. methylation data were performed with array. Sequencing data use methyl frequency and array data use beta value for the input data. Therefore, we could not directly compare the data. Instead, we compared and classified the data by 10,000 selected CpGs, which is the most variable probes that is analyzed by random forest algorithm performed by the original study team members at German cancer research center (DKFZ). Among the selected CpGs, 8546 CpGs were intersected with our data (Supplementary Data Set 1).

### CNV and SNV analysis

BWA (ver. 0.7.15) was used to align the reads to the reference genome GRCh37 (ref. ^39^). The reads were validated following the GATK (ver. 3.8.0) pipeline^15^. Somatic mutations were called with Mutect2 and germline mutations were detected with Haplotypecaller. Both mutations were filtered with 1000 g, ExAC, genomAD (allele frequency <0.01) databases. Exonic and splicing region was focused. For somatic mutation, tumor mutation burden was calculated with number of somatic mutations divided by target size (60 Mb). Copy number variation was detected with CNVkit^14^.

### Normal brain dataset

Sequencing reads and meta-data for normal postmortem human brains were downloaded through Synapse.org at accession syn12299750 (refs. ^19,20^). These are from postmortem tissue homogenates of dorsolateral prefrontal cortex gray matter approximating Brodmann area 46/9 in postnatal samples. RNA-seq libraries were constructed from high RNA quality samples using Illumina mRNA sequencing sample Prep Kit following the manufacturer’s protocol, and the final cDNA libraries were sequenced by Illumina HiSeq 2000 with 100 bp paired-end reads after multiple levels of quality controls.

### Gene expression analysis and pathway analysis

RNA reads were aligned to the reference GRCh37 by STAR aligner. Expected counts and FPKM were calculated with RSEM package (ver. 1.3.1)^18^. RNA fusion analysis was done using STAR fusion (ver. 1.4.0)^17^. We validate the fusion by removing the spanning read if it has “0” read. There were several fusions that share one gene with same spanning read counts and junction read counts. We also eliminated those fusions. If one of the fusion genes is not a protein coding gene, it is considered as non-significant fusion. Gene enrichment pathway analysis was done using the gProfiler webserver functional profiling option^21^. The Gene Ontology, KEGG and Reactome pathway databases were included as sources in the analysis. Bonferroni correction of less than 0.05 was used as significance threshold.

### RT-PCR

RT-PCR for *CLIP2-MET* fusion gene was done using cDNA made from the tissue extracted RNA using the RNA to cDNA EcoDry premix (Takara Bio, CA, USA). Normal brain RNA was used as a negative control. The primers were designed according to the breakpoint of each gene detected by the RNA fusion analysis. The Primers used are as following. The fusion gene primers were made up of *CLIP2* forward and *MET* reverse primers. RT-PCR was done using a BioRad C1000 thermal cycler. The annealing temperature for the primers were 58 °C for *CLIP2* and *CLIP2-MET* fusion and 56 °C for *MET*. Primer sequences used are as follows: CLIP2 forward: TGCAGGACAAGCTGAACAAG, CLIP2 reverse: CCTGGCTGATGAGGACTAGC; MET forward: GGTTTTTCCTGTGGCTGAAA, MET reverse: GCTACTGGGCCCAATCACTA. All blots derive from the same experiment and were processed in parallel (un-cropped images of all blots in Supplementary Information).

### Immunohistochemistry

Formalin-fixed paraffin-embedded (FFPE) tissue blocks were cut into 3-μm-thick slices and underwent immunohistochemistry. Tissue sections were stained with anti-Neu-N (Milipore, Temecula, USA, 1:500), anti-Synaptophysin (Novocastra, Newcastle, UK, 1:200), anti-CD34 (DAKO, Glostrup, Denmark, 1:200), anti-ATRX (Sigma-Aldrich, St Louis, USA, 1:200), anti-Olig2 (Cell Marque, Rocklin, USA, 1:500), anti-GFAP (DAKO, Glostrup, Denmark, 1:200), anti-Ki67 (DAKO, Glostrup, Denmark, 1:100), and anti- c-MET (Ventana Medical System, Tucson, USA, ready to use). Immunohistochemical staining was carried out using a standard avidin–biotin peroxidase method. The appropriate positive controls were used and primary antibodies were omitted as negative controls.

### Reporting summary

Further information on research design is available in the Nature Research Reporting Summary linked to this article.

## Supplementary information

Supplementary Information

Supplementary Data 1

Reporting Summary

## Data Availability

The datasets generated during the current study are available in the NCBI GEO (accession number: GSE142668). Supplementary Data Set 1 include genes with copy number alteration in chromosomes 1 and 7, somatic mutations found in the tumor, differentially expressed genes in the tumor compared with normal brain, pathway analysis using differentially expressed genes, CpG site list used for methylation classification.
