# Supplementary figures and images for: A glioneuronal tumor with CLIP2-MET fusion
Source: NPJ Genom Med. 2020 Jun 3;5:24. doi: 10.1038/s41525-020-0131-6 (PMC7270112; doi:10.1038/s41525-020-0131-6)

# Rt-PCR gel image – Original

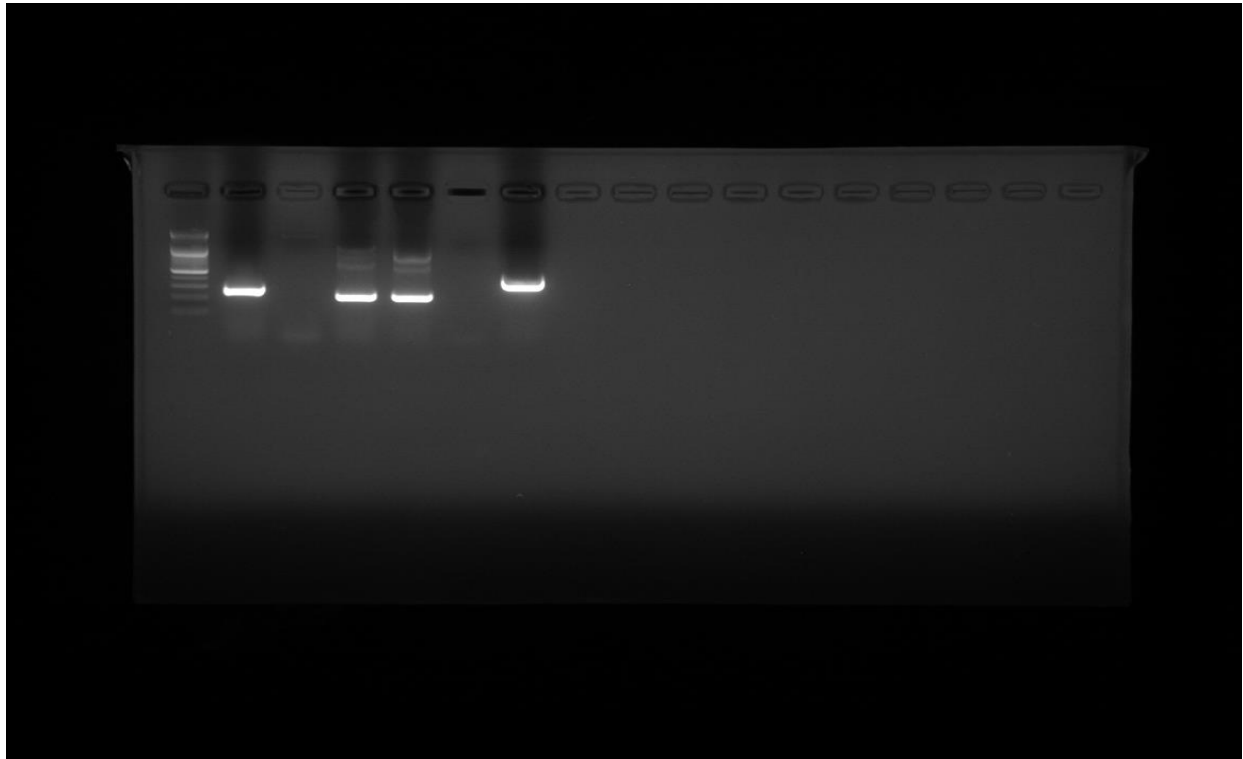

**Rt-PCR gel image – Original inverted**

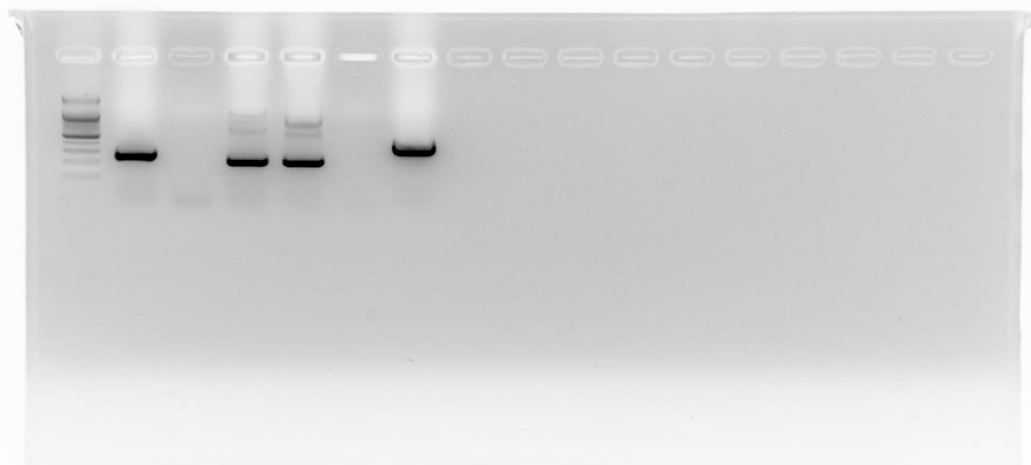

Supplement: Supplementary file 1 — Supplementary Information [file 41525_2020_131_MOESM1_ESM.pdf]
